# Addressing the workforce capacity for public health surveillance through field epidemiology and laboratory training program: the need for balanced enhanced skill mix and distribution, a case study from Tanzania

**DOI:** 10.11604/pamj.2020.36.41.17857

**Published:** 2020-05-27

**Authors:** Susan Fred Rumisha, Rogath Saika Kishimba, Ahmed Abade Mohamed, Loveness John Urio, Neema Rusibayamila, Muhammad Bakari, Janneth Mghamba

**Affiliations:** 1 National Institute for Medical Research, 3 Barack Obama Drive, Dar es Salaam, Tanzania; 2 Tanzania Field Epidemiology and Laboratory Training Program, Dar es Salaam, Tanzania; 3 Ministry of Health, Community Development, Gender, Elderly, and Children, Dodoma, Tanzania

**Keywords:** Tanzania Field Epidemiology and Laboratory Training Program, workforce, enhanced-skill mix, skill gap, public health surveillance system, Tanzania

## Abstract

**Introduction:**

Skill mix refers to the range of professional development and competencies, skills and experiences of staff within a particular working environment that link with specific outcome while responding to client needs. A balanced skill-mix and distribution of core human resources is important to strengthen decision-making process and rapid responses. We analysed graduates´ information of the Tanzania Field Epidemiology and Laboratory Training Program (TFELTP) between 2008-2016, distribution of skill-mix and the surveillance workforce-gaps within regions.

**Methods:**

Trainees´ data of nine cohorts enrolled between 2008 and 2016 were extracted from the program database. Distribution by sex, region and cadres/profession was carried out. An indicator to determine enhanced-skill mix was established based on the presence of a clinician, nurse, laboratory scientist and environmental health officer. A complete enhanced skill-mix was considered when all four were available and have received FELTP training.

**Results:**

The TFELTP has trained 113 trainees (male=71.7%), originated from 17 regions of Tanzania Mainland (65.4% of all) and Zanzibar. Clinicians (34.5%) and laboratory scientists (38.1%) accounted for the most recruits, however, the former were widely spread in regions (83% vs. 56%). Environmental health officers (17.7%) were available in 39% of regions. The nursing profession, predominantly lacking (6.2%) was available in 22% of regions. Only two regions (11.7%) among 17 covered by TFELTP presented complete skill-mix, representing 7.7% of Tanzanian regions. Seven regions (41%) had an average of one trainee.

**Conclusion:**

The TFELTP is yet to reach the required skill-mix in many regions within the country. The slow fill-rate for competent and key workforce cadres might impede effective response. Strategies to increase program awareness at subnational levels is needed to improve performance of surveillance and response system in Tanzania.

## Introduction

A functional public health surveillance system is essential for improving response to public health events. One of the qualities of an effective system is competent and highly skilled human resources [1-6]. Field Epidemiology Training Programs (FETPs) have been introduced in many developing countries in sub-Saharan Africa and Asia [3,4,7-10]. These programs were designed to create a pool of highly skilled health workers who could effectively strengthen public health surveillance and efficiently respond to public health threat including outbreaks [1-3,8,10]. The program was modelled after CDC Epidemic Intelligence Service (EIS) program that has been running for decades [11]. The training strategy adopted by FETP is competency-based where trainees are coached while providing services. The trainees spend over 70% of their training time in the field working and 30% of the time in class doing didactic. Since the inception of these programs, there has been remarkable improvement in disease detection and response in sub-Saharan Africa [4,5,^12^,^13^]. In 2008, Tanzania Ministry of Health started Field Epidemiology and Laboratory Training Program (TFELTP) in recognition of the need to strengthen epidemiological and surveillance capacity in the country [3]. Since then, several cadres including clinicians, laboratorians, environmental health officers, nurses, pharmacists and vetenerians were enrolled. The response to have a wide range of professions was important to strengthen management capacity to address both communicable and non communicable diseases [3]. To-date, a number of personnel with varying professions and disciplines have been trained in the program [3,6]. The program is a partnership between the Ministry of Health, Community Development, Gender, Elderly and Children, Muhimbili University of Health and Allied Sciences, the National Institute for Medical Research and international partners. Partners include Centers for Disease Control and Prevention - U.S. President's Emergency Plan for AIDS Relief, United States Agency for International Development - President's Malaria Initiative, International Association of National Public Health Institutes, World Bank and African Field Epidemiology Network (AFENET) [3]. Trainees of TFELTP are drawn from various regions of Tanzania with entry requirements covering a wide range of disciplines with two (2) years of experience [3,14,^15^]. That inclusion ensures continuum of mix skillful workforce needed for implementation of the Integrated Disease Surveillance and Response (IDSR) system [1,4,^10^,^14^-^18^]. During and after the training, trainees and residents are involved in supervision and response to outbreaks in several parts of the country and internationally [5,13,^14^]. Thus, this training model and the design of the TFELTP, adopted from EIS [11] provides a comprehensive and extensive learning opportunities through exposing trainees to long field work experiences where challenges, multisectoral interactions, cross-sectoral collaboration and other strategies for implementing surveillance activities are acquired [1,3,9,15]. Having TFELTP graduates who have mixed skillful is thus important, as they all provide a wider scope of varying knowledge, improve the decision-making processes, and understandability of possible consequences and risks that are necessary for instituting rapid and efficient response actions [17,19-21]. A balanced and a well distributed skill mix in human resources also ensures effective implementation of IDSR [16,18,^20^-^22^]. Regardless of the evidence that the presence, coverage and distribution of FELTP trainees at subnational levels in number of countries [4,5,10] have improved disease surveillance and response to outbreaks and public health events, and management of health care systems [9]; there is no information on how the programs have managed to reach the required surveillance skill mix at subnational level. This paper analyses information from the Tanzania FELTP program, aim to document the distribution of TFELTP trainees, in terms of professional characteristics, and also quantifies the skill-mix that has been established at regional level since commencing of the program in 2008. The paper then quantifies the skill-mix gap required for performing core surveillance actions and, explore strategies to fill the gaps. Lessons learnt from this analysis are relevant not only to field epidemiology training programs but also to the departments responsible for implementing IDSR strategy particularly in guiding strengthening surveillance workforce.

## Methods

**Design and data sources:** data used for this analysis include information of trainees enrolled in nine cohorts between 2008 and 2016. The database, maintained by the TFELTP program administrator, contained information on the characteristics of trainees and graduates and is updated each time a new cohort is recruited. Details extracted included trainees´ sex, professional background and place of (origin) work. For data confidentiality, personal and identifiable information such as name and administrative titles were not used. Dataset used for this study is available upon reasonable request from the Tanzania Field Epidemiology and Laboratory Training Program Director.

**Data analysis:** obtained data were summarized to obtain distribution and patterns of all variables of interest such as sex, region of origin before joining the program and profession. An indicator to determine enhanced-skill mix and skill gaps within core surveillance workforce was established based on the presence of core cadres supposed to form a public health emergency management committee [23] who received FELTP within a region. This includes a medical doctor (clinician), a public health nurse, a laboratory personnel (technician or technologist) and an environmental health officer. The indicator ranged between zero (0) and four (4). The value of four indicates complete or correct mix and was considered when all of these cadres were available and have received TFELTP training while a value of zero (none) indicates poor or no enhanced-skill mix. A value of two (2) was taken as an average performance. The performance of each region is presented and discussed. Regional coverage of FELTP training for the core surveillance cadres/professions in Tanzania for the decade when the program has been in operation was also discussed. Results are presented using Tables and Figures. Maps were developed to depict regional distribution of the defined indicators and to study the spatial variation within surveillance workforce. Data were managed in Microsoft Excel and maps were produced using ArcGIS 9.2 GIS Software.

**Ethics approval and consent to participate:** permission to get access to the TFELTP database was sought from the program director who is also the assistant director for Epidemiology and Disease Control Section of the Ministry of Health, Community Development, Gender, Elderly and Children, Tanzania. Information were extracted by the program administrator where all personal information that could link to the identification of the trainee were removed. The data were managed considering confidentiality and anonymity.

## Results

Since the inception of TFELTP in 2008, the program has enrolled a total of nine cohorts and trained 113 trainees (112 from Tanzania and 1 from Zambia). There was a high difference between the proportion of males and females [81; (71.7%) vs 32; (28.3%)] respectively, with male: female ratio of 2.5:1 trainees enrolled in the program. During the first five years (2008-2012) the average number of trainees per cohort was 12 and rose to 14 in the recent years.

**Figure 1:**
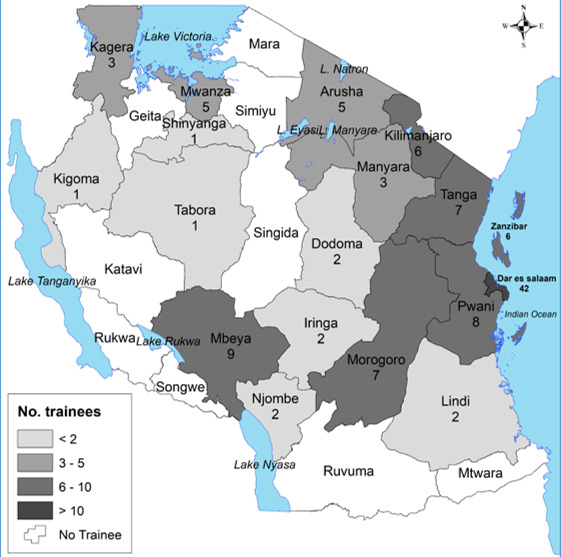
Distribution of number of TFELTP trainees by original place of work, 2008-2016

**Region of origin:** trainees originated from 17 regions of Tanzania Mainland representing 65.4% of all regions and a few were from Zanzibar (Figure 1). Dar-es-Salaam region had the highest number of trainees followed by Mbeya and Pwani regions. Regions with the lowest number of trainees include Kigoma, Shinyanga and Tabora. Regions (with geographical zones) that have not yet trained any staff include Mtwara and Ruvuma (Southern), Singida (Central), Rukwa and Katavi (Western), Songwe (Southern Highlands), and Mara, Simiyu, and Geita (Lake).

**Figure 2:**
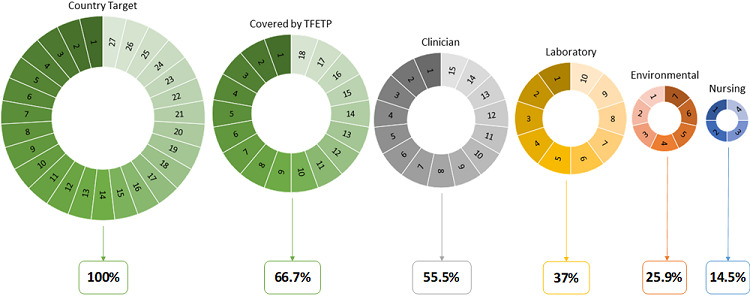
Target and coverage of FELTP training for the core surveillance cadres/professions in Tanzania, 2008-2016 (labels indicate the number of regions)

**Professional coverage:** trainees´ professional background included dental surgeons, environmental health, laboratory science, doctor of medicine (clinician), nursing, nutrition and pharmacy (Table 1). Sex distribution in these professions indicated high inclusion of males in all recruited professions. The professions with low numbers included those from nutrition (1 trainee, female), pharmacy (1 trainee, male) and dental surgeons (2 trainees, females) (Table 1). Distribution of the professions in the regions is presented in Table 2. Medical epidemiologist (clinician) was a major professional skill covered and widely spread in most regions (83%, 15/18 regions). Despite a large number of laboratory epidemiologists, they originated from only 10 regions (56%). Low (39%) regional distribution pattern was observed for environmental health personnel. Out of 20 trainees whom originated from seven regions, 55% (n=11) came from Dar es Salaam region alone. The nursing profession was poorly represented and originated only from four regions (22%) (Table 2). In Figure 2, number and proportions of targeted regions (all 26 in Tanzania including Zanzibar), those covered by the TFELTP and where the four core professions exists are presented to visualize the extent of the gap in the surveillance workforce.

**Table 1:** Professions of the Tanzania FELTP trainees since 2008-2016

Profession	Female	Male	Total
	N (r%)	N (r%)	N (c%)
Dental Surgeon	2 (100)	0 (0)	2 (1.8)
Environmental Health	7 (35)	13 (65)	20 (17.7)
Laboratory Science	9 (20.9)	34 (79.1)	43 (38.1)
Doctor of Medicine (Clinician)	11 (28.2)	28 (71.8)	39 (34.5)
Nursing	2 (28.6)	5 (71.4)	7 (6.2)
Nutrition	1 (100)	0 (0)	1 (0.9)
Pharmacy	0 (0)	1 (100)	1 (0.9)
Total	32 (28.3)	81 (71.7)	113

r%= Row percentage; c%= Column percentage

**Table 2:** Professions distribution of TFELTP trainees by regions indicating presence of the core surveillance cadres, 2008-2016

Region	Professions required for Surveillance							
	Core				Other			
	MD	LSc	EH	N	DS	Nutrition	Pharmacy	Total
Arusha	1	0	2	2	0	0		5
Dar-es-Salaam	12	17	11	0	2	0	0	42
Dodoma	2	0	0	0	0	0	0	2
Iringa	1	0	1	0	0	0	0	2
Kagera	3	0	0	0	0	0	0	3
Kigoma	1	0	0	0	0	0	0	1
Kilimanjaro	0	6	0	0	0	0	0	6
Lindi	1	1	0	0	0	0	0	2
Manyara	3	0	0	0	0	0	0	3
Mbeya	2	1	2	3	0	0	1£	9
Morogoro	2	5	0	0	0	0	0	7
Mwanza	3	2	0	0	0	0	0	5
Njombe	0	1	1	0	0	0	0	2
Pwani	2	5	0	1	0	0	0	8
Shinyanga	0	0	1	0	0	0	0	1
Tabora	1	0	0	0	0	0	0	1
Tanga	2	2	2	1	0	0	0	7
Zanzibar	3	2	0	0	0	1£	0	6
Total trainees	39	42	20	7	2	1	1	112
Regions with trainee	15	10	7	4	1	1	1	
% of regions with trainees	83%	56%	39%	22%	6%	6%	6%	

Key: MD=Doctor of Medicine (clinicians), LSc=Laboratory Science (technicians/technologists), EH=Environmental Health, N=Nursing, DS=Dental Surgeon; £Uncommon in FETP

**Figure 3:**
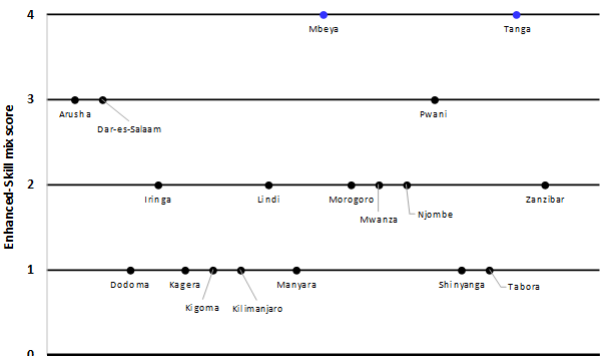
Scores for enhanced-skill mix for the surveillance workforce by regions indicating number of core staff who received Field Epidemiology and Laboratory Training, Tanzania

**Figure 4:**
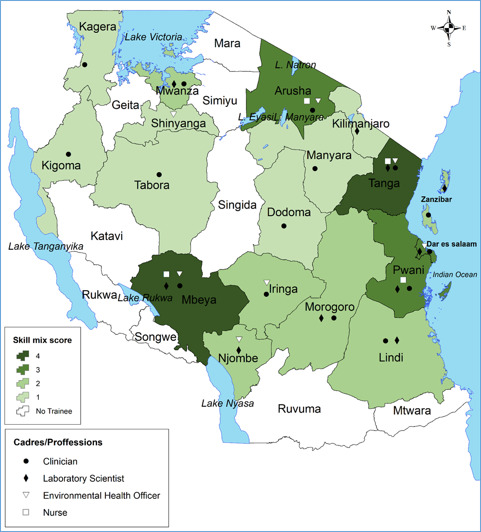
Map showing the spatial variations in enhanced skill-mix score and availability of core surveillance staff received Field Epidemiology and Laboratory Training in regions of Tanzania

**Skill mix:** estimated regional enhanced skill-mix scores are presented in Figure 3. Only Tanga and Mbeya regions presented complete skill mix. Arusha, Dar es Salaam and Pwani regions had a score of 3 indicating that among the core four cadres, 3 already received FELTP training. Six regions (35.3%) had an average score while seven regions (38.9%) were below the average score (only one FELTP trainee) (Figure 3). Spatial variations of the enhanced skill-mix and availability of the core cadres/professions by regions is shown in Figure 4.

## Discussion

We present here information on the status of the Tanzania FELTP in filling up the workforce for public health surveillance system at subnational levels. The program has successfully enrolled nine cohorts with over 99% of the candidates graduate each year. About two-third of the graduates are currently working at public sectors serving at districts, regions and national levels, a fifth are in the private sector including non-governmental organisations, and around three-percent in bilateral organizations (Source: TFELTP Documentation, Unpublished materials). This incredible achievement has been observed in other FETPs [5-7] and other applied epidemiology trainings [24]. The model of training used by FELTP provides opportunity to acquire practical skills gained at field, conducting analysis and practical involvement in surveillance activities [3,25]. “Learning by doing,” maintains a spontaneity and significance that are essential to addressing the public health challenges [9,25] that discerns the abilities, capacities and capabilities of FELTP trainees during implementation. Most of TFELTP trainees were observed to be clinicians, laboratory scientists and environmental health officers. Similar tendency has been seen in other FETPs including Kenya [26], Jordan [27], India [7] and many more including EIS [11]. Nevertheless, effective implementation of surveillance activities requires strength in all surveillance components including detection, diagnosis, treatment, laboratory investigation and reporting, utilizing the information (for evidence-based decision) [23], which demand expertise from other disciplines. Results of this analysis indicate that some crucial skills such as veterinarians, data analysts, statisticians and computer programmers are weakly represented in the program. This pattern might be driven by selection criteria, (original) requirements (e.g. qualification with a Bachelor´s degree which was not always available in some professions) and national priorities, on the other hand, poor awareness of the benefits of such a training to other disciplines might be a contributing factor [26,28]. As the program grows and considering surveillance technical demands and capacity requirements at the subnational level, there is a need to establish strategies to improve awareness through sharing successes and lessons learned to create demand.

Skilled workforce and well distributed from national to subnational levels, allows prompt response to public health events [1,4,12,16,19]. A high proportion of trainees originated from Dar es Salaam region, where the course is conducted [3], and ministries and national departments were formally located (currently moved to Dodoma). It is convenient for someone residing in Dar es Salaam to attend classes and lectures without incurring high living expenses, particularly those related to accommodation and food. Closeness and convenience might drive the awareness to the program, number of applications and later those recruited. This may explain also a large number of trainees from regions closer to Dar es Salaam including Pwani, Morogoro and Tanga. A scenario of regions like Mbeya, Arusha and Kagera, located far from Dar es Salaam, with a good number of trainees can be interpreted as leadership understanding on the demand and value of the FELTP program. Contrariwise, technical challenges such as poor and unreliable internet connectivity could deter and discourage applications from remote regions. Application to TFELTP requires internet. Applicants from urban and sub-urban regions may be best-placed to access good internet connection than those in upcountry. That being the case, it is important for the program to monitor such patterns and make strategies to reach applicants from remote regions, newly introduced regions, regions with financial hardships, with poor connectivity and those that have not been covered at all [4,26]. On the other hand, deliberate recruitment of national level staff may be done to strengthen the central unit, nevertheless, decentralization of responsibilities, skills and capacities at sublevels is vastly needed [26]. Impact and importance of skill mix in clinical outcomes have been studied by a number of scholars [29-32]. It has proven that, skill-mix decisions save more lives, guide proper allocation of resources and create cost-effective systems [32-34]. In disease surveillance and response, we expect optimal efficiency when responsible staff have similar capacity, understand roles and responsibilities and the interactions needed in expertise. Within 8-years of the TFELTP, our analyses observed gaps in enhanced-skill mix in more than half of the regions indicating a slow rate in filling complete skill mix throughout the country. Tailored strategies such as introduction of short-term training frontline public health workers might fill the observed gap [12,26,35]. In addition, there is a need to establish financial support from local sources (regions and districts health budget) for those interested to join the course. Some provinces in South Africa have been reported to pay for their staff to attend FELTP [28].

FELTP was designed to provide competency-based training for those involved in disease surveillance and response [1-3]. Most countries in sub-Saharan Africa had adopted and have been implementing IDSR strategy for about two decades [36]. Core aims of IDSR include training, which makes FELTP an important engine to the success of the strategy. In a recent review on challenges on implementation of IDSR, inadequate training and uneven resources (human and financial) were mentioned among the main concerns that affect almost all IDSR functions [37]. Proposed strategies to overcome this include institutionalization of IDSR training in regular public health curricula [37,38]. The FELTP advanced and intermediate courses provide an opportunity to cover this knowledge and skill gap at both hands, epidemiology and laboratory capacities, that can be extended to those working in the lower levels via a cascade model using FELTP alumni. Findings from this study have highlighted the status of enhanced skills at different professions and regions. Triangulating these results with the observed challenges in implementing IDSR, provide a relevant guidance to TFELTP on how to prioritize its focus in strengthening and filling the skill gap to maximize effectiveness of surveillance system [14,37]. Although our approach managed to map existence of enhanced skill-mix required to perform surveillance activities in regions, this study also face some limitations. First, is the assumption that having trained staff translates to competence in performance, which is not necessarily the case. However, with the scarcity of health human resources, increasing the number of health workers with acquired field and practical enhanced-skills is a critical stage in strengthening the surveillance systems and outcomes of disease outbreak responses. Secondly, the data was analysed at regional level, which in the health system structure, act as advisory and supervisory body and not day-to-day implementation level. It is possible to mask relevant findings that could be observed if data were disaggregated at fine spatial scales. A retrospective longitudinal analysis on performance of surveillance indicators at district level is highly recommended.

## Conclusion

In summary, the Tanzania FELTP has strengthened the skills, technical knowhow and capacities of surveillance workforce in the country. However, the program is yet to reach the required skill mix ratio in many regions in the country, despite its establishment in 2008. The slow fill rate for competent workforce might impede effective response. The program should take initiatives to involve more local stakeholders, at subnational health authorities and to create awareness on benefits of the program to improve applications, sustainability and maintain equitably distribution of skilled workforce hence performance of surveillance system in Tanzania.

### What is known about this topic

- There is a clear evidence on improvement on effectiveness of public health surveillance systems in countries with established FELTPs;
- Trainees from the FELTPs have been a strong hold for the epidemiological aspects of many countries indicating competence and enhanced skills when responding to public emergencies.

### What this study adds

- A clear need to evaluate and monitor performance of the FELTPs on strengthening and creating a sustainable public health workforce;
- Highlights gaps, and emphasizes on need to strengthen networks, continuing awareness of the programs and importance of skill-mix in surveillance workforce;
- Introduce an easy tool which can be linked with other aspects to monitor effectiveness of surveillance system in countries.

## Competing interests

The authors declare no competing interests.
